# The Prevalence of and Factors Associated with Neck, Shoulder, and Low-Back Pains among Medical Students at University Hospitals in Central Saudi Arabia

**DOI:** 10.1155/2017/1235706

**Published:** 2017-11-07

**Authors:** Abdulrahman D. Algarni, Yazeed Al-Saran, Ahlam Al-Moawi, Abdullah Bin Dous, Abdulaziz Al-Ahaideb, Shaji John Kachanathu

**Affiliations:** ^1^Department of Orthopedic Surgery, King Saud University, Riyadh, Saudi Arabia; ^2^College of Applied Medical Sciences, King Saud University, Riyadh, Saudi Arabia

## Abstract

**Aim:**

The study aim was to determine the prevalence of neck, shoulder, and low-back pains and to explore the factors associated with musculoskeletal pain (MSP) among medical students at university hospitals in central Saudi Arabia.

**Method:**

This cross-sectional study was conducted at a government institution using an online self-administered, modified version of the Standardised Nordic Questionnaire in the English language.

**Results:**

A total of 469 students responded to our survey. The prevalence of MSP in at least one body site at any time, in the past week, and in the past year was 85.3%, 54.4%, and 81.9%, respectively. Factors significantly associated with MSP in at least one body site at any time were being in the clinical year (*P* = 0.032), history of trauma (*P*  =  0.036), history of depressive symptoms (*P* < 0.001), and history of psychosomatic symptoms (*P* < 0.001). On multivariable regression analysis, factors associated with MSP were history of trauma (*P* = 0.016) and depressive (*P* = 0.002) or psychosomatic symptoms (*P* = 0.004).

**Conclusion:**

MSP among Saudi medical students is high, particularly among those in the clinical years and those with history of trauma and with depressive or psychosomatic symptoms. Medical institutions should be aware of this serious health issue and preventive measures are warranted.

## 1. Introduction

Musculoskeletal disorder (MSD) is a health hazard that affects a significant majority of individuals, particularly those in the workforce. The 2014 report on the global disability burden of MSD ranked it sixth with respect to disability-adjusted life years [1, 2]. Low-back and neck pains were listed among the diseases with the greatest number of years lived with disability. The prevalence of MSD, particularly those causing musculoskeletal pain (MSP) in the neck, shoulder, and lower back, has been reported from various sectors of the society. MSP is very common among Norwegians, affecting 75%–80% of the population [3]. The prevalence of work-related MSP involving the neck, shoulder, and upper back was reported to be between 35% and 45% among midwives, nurses, and doctors [4]. A higher prevalence of MSP (50%–93%) was even reported among dental personnel with the shoulder being the predominantly painful area [5, 6].

Similarly, several reports have described the occurrence of MSP among college students [7–10]. The prevalence of complaints of MSP in any body site was 37% among X-ray technology students [7]. Among music school students, the reported prevalence rate was between 38.2% (lower back pain) and 60.4% (neck pain) [8]. Among dental hygiene students, the prevalence of neck pain (64%), lower back pain (57%), and shoulder pain (48%) was high [9]. The reported prevalence of MSP among medical students was between 45.7% and 65.1% [10].

The onset of MSP is believed to be triggered by certain factors. These include frequent repetitive movements of a particular body part [11, 12], certain positions like prolonged standing or sitting [13, 14], and sports or occupational activities [5]. Increased body mass index (BMI) is strongly associated with MSP involving the neck, shoulder, lower back, and foot [15]. There were also significant associations found between female sex, older age, and smoking with neck pain, but not with low-back pain [16–18]. Some studies have associated long hours of computer use to MSP [19, 20]. People with MSP may experience poor quality of life and suffer from depression and various psychosomatic symptoms [5], and as such, we also deemed these factors worthy of inclusion in our investigation.

All undergraduate and postgraduate medical education in Saudi Arabia is conducted in the English language. To obtain a bachelor degree of medicine and surgery, the candidate has to spend at least 7 years. The first year is a preparatory year outside the medical college during which the candidate has to obtain a certain Grade Point Average (GPA) and a certain score in an English-proficiency examination, either the International English Language Test System (IELTS) or the Test of English as a Foreign Language (TOEFL) to join the medical college. The 7th (final) year is an internship year spent at hospitals affiliated to the medical college and is a requirement for graduation. The five years in between are spent at the medical school starting from academic year 1 to academic year 5 and it is the student population in this period that is of interest to our current research. The academic years 1, 2, and 3 are preclinical years and the academic years 4 and 5 are clinical.

The main aim of the study was to determine the prevalence of neck, shoulder, and low-back pains and to explore factors associated with MSP among medical students at university hospitals in central Saudi Arabia. Furthermore, we assessed the impact of MSP on the students' quality of life.

## 2. Methods

This cross-sectional study was conducted among all medical students (2357) enrolled at the College of Medicine at King Saud University and King Saud Bin Abdulaziz University for Health Sciences, Saudi Arabia, during the period of June to December 2014. This study used an online self-administered questionnaire in the English language. The questionnaire was a modified version of the Standardised Nordic Questionnaire that was adapted for use in medical students and validated by previous authors [10, 21, 22]. We used this questionnaire to allow comparison of our findings of MSP in medical students with others. The questionnaire sought information on sociodemographic characteristics and factors such as exercise, caffeine consumption, smoking, any history of trauma, depressive or psychosomatic symptoms, and specific questions pertaining to musculoskeletal pain occurrences in the neck, shoulders, and low-back. The questionnaire was pretested on twenty students before distributing to the participants to ensure understandability of the questions by nonnative English-speaking students. The pilot study showed a good understanding and a clear comprehension in the responses provided. E-mails were sent to all medical students (first year to fifth year) with a cover letter explaining the purpose of the study and ensuring their anonymity. The survey was conducted using the online survey software from Google Drive (docs.google.com). Ethical approval was obtained from our Institutional Review Board.

All statistical analyses were performed with SPSS statistical software version 22 (IBM Corp., Armonk, NY, USA). Categorical variables are presented as proportions. Continuous variables are presented as means with standard deviations if they are normally distributed and medians with 25th–75th percentile if the distribution is nonparametric. Chi Square or Fisher's exact test was used to compare categorical variables, as appropriate. Student's *t*-test was employed to compare continuous variables that have normal distribution while Mann–Whitney test was used for variables with nonparametric distribution. Statistical significance was based on two-sided *P* values less than 0.05.

Multivariable analysis to evaluate predictors of musculoskeletal pain at any time (dependent variable) was performed using the binary stepwise logistic regression model. We determined a priori a set of predictors based on their clinical or biological importance and also based on the *P* value (*P* < 0.1) of potential confounders from the univariate analysis. Model discrimination was evaluated with the area under the ROC curve (*C*-statistics, with 95% Confidence Interval). The model calibration was evaluated with the Hosmer–Lemeshow goodness-of-fit statistic where a *P* value > 0.05 was indicative of adequate calibration.

We validated the final model with bootstrapping using 1000 resamples. Finally, we evaluated collinearity in the model (high correlation between predictors) using the variance inflation factor (VIF). We defined a significant collinearity as a VIF > 2.5.

## 3. Results

A total of 469 students responded to our survey. The response rate was 19.9%. Mean age was 21.4 ± 1.3 years ranging from 19 to 29 years. There were 185 (39.64%) males and 284 (60.6%) females. There were 247 (52.7%) in the preclinical years and 222 in the clinical years (47.3%). Mean BMI was 24.3 ± 5.7 (range: 15.2 to 55.8). Majority of the participants (*n* = 372, 79.3%) exercised regularly; 321 (68.4%) exercised by walking or running. Thirty-six (7.7%) of the participants used tobacco.  Table 1 presents the demographic characteristics of all survey participants. The prevalence of MSP (in at least one body site) was 85.3% (*n* = 400) at any time. The prevalence of MSP (in at least one body site) in the past week was 54.4% (*n* = 255) and 81.9% (*n* = 384) in the past year. The distribution of pain during those periods based on site is summarised in Table 2.

### 3.1. Neck Pain

The prevalence of neck pain was 24.1% (*n* = 113) in the past week and 56.5% (*n* = 265) in the past year. There were 17 participants (3.6%) who claimed to have sustained trauma to the neck; two (0.4%) were hospitalised because of neck pain. Sixty-two (13.2%) of the participants claimed that neck pain affected their student life, in the form of frequent absences from school and difficulties in performing their usual duties. Thirty-one (6.6%) had been seen by a physician, physiotherapist, or other health professionals for neck pain. There were 125 (26.7%) participants who claimed that neck pain occurred after a specific movement of the body and 190 (40.5%) claimed that neck pain occurred after prolonged sitting.

### 3.2. Low-Back Pain

The prevalence of back pain was 40.5% (*n* = 190) in the past week and 67.0% (*n* = 314) in the past year. Seventy participants (14.9%) claimed to have sustained some form of trauma to the back; 14 (3.0%) were even hospitalised because of back pain. There were 116 (24.7%) who claimed that back pain affected their life, and 63 (13.4%) consulted a physician, physiotherapist, or other health professionals because of back pain. Thirty-three participants (7.0%) claimed that back pain was accompanied sciatica. There were 201 (42.9%) participants who claimed that back pain occurred after prolonged standing. There were 127 (27.1%) participants who always carried a backpack, 202 (43.1%) sometimes carried a backpack, and 140 (29.9%) who never carried a backpack. There were 270 (57.6%) students who studied on a chair and table, 189 (40.3%) on the floor, and 10 (2.1%) lying on the couch.

### 3.3. Shoulder Pain

The prevalence of shoulder pain was 25.6% (*n* = 120) in the past week and 45.6% (*n* = 214) in the past year. Of this, 34 (7.2%) claimed to have sustained trauma to their shoulder, and 7 (1.5%) were hospitalised because of shoulder pain. Fifty-three participants (11.3%) claimed that shoulder pain affected their life and performance of daily duties. Thirty-two participants (6.8%) consulted a physician, physiotherapist, or other allied health professionals. There were 103 (22.0%) students who claimed to participate in activities that required lifting heavy load overhead. There were 118 (25.2%) students that participated in activities that included vibration or repetition of certain movements. There were also 136 (29.0%) students who often maintained an awkward posture, however; it was not significantly associated with MSP (*P* = 0.245).

### 3.4. Factors Associated with MSP in at Least One Body Site at Any Time

Tables 3 and 4 present the factors associated with MSP in univariate and multivariable regression analysis. The overall prevalence of MSP regardless of the time of occurrence was not significantly associated with sex (OR 1.22, 95% CI 0.73–2.01, *P* = 0.458). There was a significant association between the prevalence of MSP and being in the clinical year (OR 1.38, 95% CI 0.82–2.32, *P* = 0.032). Exercise was not significantly associated with MSP (*P* = 0.515). The consumption of caffeine was not significantly associated with MSP (*P* = 0.713). Smoking was not significantly associated with MSP (*P* = 0.747). The duration of computer use was not significantly associated with MSP (*P* = 0.719). There was no significant association between MSP and age (*P* = 0.994) or BMI (*P* = 0.58). History of trauma was significantly associated with any MSP at any time (OR 2.1, 95% CI 0.96–4.53, *P* = 0.036). Trauma was significantly associated with an increased prevalence of neck (*P* = 0.015), low-back (*P* = 0.008), and shoulder pains (*P* = 0.044). There were 275 (58.6%) participants who claimed that they experienced depressive symptoms related to their studies as medical students. There were 180 (38.4%) participants who experienced psychosomatic symptoms in the form of numbness, tingling, or weakness in any part of their body. MSP was strongly associated with the depressive symptoms that student suffered related to their studies as medical students (OR 2.93, 95% CI 1.73–4.98, *P* < 0.001). MSP was also significantly associated with the psychosomatic symptoms (OR 2.69, 95% CI 1.37–5.27, *P* = 0.004). On multivariable logistic regression analysis, factors associated with MSP in at least one body site at any time were history of trauma (OR 1.92, 95% CI 0.87–4.23, *P* = 0.016) and depressive (OR 2.35, 95% CI 1.36–4.67, *P* = 0.002) or psychosomatic symptoms (OR 2.69, 95% CI 1.37–5.27, *P* = 0.004). The total model was significant (area under the ROC curve = 0.69, 95% CI 0.63–0.749, *P* < 0.001).

## 4. Discussion

To the best of our knowledge, there are only few reports in the literature that specifically address the occurrence of MSP in undergraduate medical students. Alshagga et al. [10] have studied the prevalence of MSP among Malaysian medical students. The authors found that 45.7% and 65.1% of all students had at least one site of MSP in the past week and in the past year, respectively. Among Chinese medical students, MSP was reported most commonly in the lower back with a prevalence rate of 46.9% in the past week and 67.6% in the past year [22]. In the present study, the prevalence of MSP in Saudi medical students was also high and comparable to the reported rates from the Malaysian and Chinese studies [10, 22]. We found that 85.3% of all the students had MSP in at least one body site at any time, 54.4% in the past week, and 81.9%, in the past year. Indeed, this high prevalence rate of MSP found in our medical students was higher than the reported rates in midwives, nurses, doctors, and dental professionals [4–6]. This finding further affirms that a high prevalence of MSP is seen among medical students and not only among individuals involved in strenuous physical work. This area requires further research to explore whether cultural, educational, or dietary factors could contribute to this high prevalence of MSP. Binsaeed et al. [23] have reported within our study population a high prevalence of vitamin D deficiency, which has been shown to be associated with MSP by several authors [24–26].

In our study, the significantly higher prevalence of MSP found among medical students in the clinical years compared to their peers in the preclinical years was consistent with the findings reported by Alshagga et al. [10]. Both studies confirm the strenuous training of medical students in the clinical years.

The statistical insignificance of sex, age, and BMI with respect to MSP in our study contradicts the findings reported by previous studies [15, 18]. Butterworth et al. [15] considered high BMI to be a risk factor for MSP. Similarly, Wærsted et al. [18] associated female sex and old age with the occurrence of MSP. Probably as our study population belonged to one age group (young adults) with almost similar levels of activity, the variation according to the age or sex would likely be small. The significant association between MSP and history of trauma in the present study is understandable. Individuals who had sustained a traumatic injury to the neck, shoulder, and back were at higher risk to develop MSP. The insignificant association between other physical and clinical variables such as exercise, caffeine consumption, smoking, and computer use with MSP is in contrast to the studies that associated such factors to the occurrence of MSP [5, 16, 18–20]. We propose that the relatively large sample size of 469 medical students that participated in this study, which represents the largest sample size to date, may have obviated the likelihood of these factors being associated with MSP.

The present study also showed that 13.2%, 24.7%, and 11.3% of the participants claimed that neck, low-back, and shoulder pains, respectively, affected their student's quality of life, in the form of frequent absences from school and difficulties in performing their usual activities. As a result, 58.6% of the participants claimed that they experienced depressive symptoms related to their study as medical students; therefore, the affected students may have experienced low self-esteem which reflected negatively on their performance in school and on their hospital duties. This finding offers insight into the significant impact of MSP on medical students, which needs to be addressed. Universities should take preventive measures in order to provide their students with a decent environment for successful academic life. In addition, developing and implementing corrective measures to prevent depressive symptoms and to improve quality of life of medical students are warranted. The impending health issues that may beset medical students require further attention and intervention. There is need for more longitudinal studies to determine the nature of this disorder and to institute early recognition of MSP to prevent long-term sequelae.

Although the number of participants (469) in the present study represents a sufficient sample size, the low response rate of 19.9% can be considered a weakness of the study. This might be due to the online form of questionnaire and the difficulty in tracking them. Also, adding an incentive to our questionnaire would probably have improved the response rate [27]. Difficulty in recalling the number of times having musculoskeletal pain over a year together with subjectivity in addressing the symptoms due to the nature of self-administered questionnaire is another weakness. Likewise, lacking an objective method for assessing the site and severity of pain is a challenge that requires a valid, reliable, and reproducible tool in order to solve it.

## 5. Conclusion

MSP among Saudi medical students is high, particularly among those in the clinical years and those with a history of trauma and depressive or psychosomatic symptoms. Medical school authorities should be aware of this health issue and formulate corrective measures to control MSP related to medical students.

## Figures and Tables

**Table 1:** Demographic characteristics of all participants (*n* = 469).

Variables		*N*	%
Sex			
Males		185	39.4
Females		284	60.6
Preclinical versus clinical			
Preclinical		247	52.7
Clinical		222	47.3
Academic year			
1st year		29	6.2
2nd year		101	21.5
3rd year		117	24.9
4th year		110	23.5
5th year		112	23.9
Exercise			
Yes		372	79.3
No		97	20.7
Forms of exercise			
Football		73	15.6
Basketball		12	2.6
Tennis		14	3.0
Swimming		85	18.1
Walking/running		321	68.4
Coffee			
No		1	0.2
<3 cups/week		219	46.7
>3 cups/week		249	53.1
Smoker			
Yes		36	7.7

	Mean (SD)	Range	

Body mass index	24.3 (5.7)	15.2–55.8	
Height	166.1 (9.4)	140.0–192.0	
Weight	67.6 (19.1)	39.5–192.0	
Hours of computer use	5.2 (2.9)	1–16	

**Table 2:** The prevalence of neck, shoulder, and low-back pains at any time, during the past week and the past year.

Body site	Prevalence of MSP during the past week		Prevalence of MSP during the past year		Overall prevalence of MSP at any time	
	*N*	%	*N*	%	*N*	%
Neck						
No	356	75.9	204	43.5	189	40.3
Yes	113	24.1	265	56.5	280	59.7
Shoulder						
No	349	74.4	255	54.4	240	51.2
Yes	120	25.6	214	45.6	229	48.8
Low-back						
No	279	59.5	155	33	142	30.3
Yes	190	40.5	314	67	327	69.7
Overall (at least one body site)	255	54.4	384	81.9	400	85.3

**Table 3:** Univariate analysis of factors associated with MSP in at least one body site at any time.

Variable		MSP		OR (95% CI)	*P* value
		Yes *N* (%)	No *N* (%)		
Sex					
Male		155 (83.8)	30 (16.2)		
Female		245 (86.3)	39 (31.7)	1.22 (0.73–2.01)	0.458
Preclinical versus clinical					
Preclinical		206 (83.4)	41 (16.6)		
Clinical		194 (87.4)	28 (12.6)	1.38 (0.82–2.32)	0.032
Exercise					
Not at all		81 (83.5)	16 (16.5)		0.515
Occasional		81 (82.7)	17 (17.3)		
Regular		238 (86.9)	36 (13.1)		
Coffee					
Not at all		1 (100)	0 (0.0)		
<3 cups/week		184 (84)	35 (16)		
>3 cups/week		215 (86.3)	34 (13.7)		0.713
Smoking					
Yes		32 (88.9)	4 (11.1)		0.747
No		367 (85)	65 (15)		
History of trauma					
Yes		86 (91.5)	8 (8.5)		
No		314 (83.7)	61 (16.3)	2.1 (0.96–4.53)	0.036
History of depressive symptoms					
Yes		250 (90.9)	25 (9.1)		
No		150 (77.3)	44 (22.7)	2.93 (1.73–4.98)	<0.001
History of psychosomatic symptoms					
Yes		168 (93.3)	12 (6.7)		
No		232 (80.3)	57 (19.7)	3.44 (1.79–6.6)	<0.001

	Yes (Median, IQR)	No (Median, IQR)			

Age	398 (22.84, 6.22)	69 (23.39, 5.23)			0.944
Body mass index	398 (21, 2)	69 (21, 1)			0.58
Computer use (hours/day)	398 (5, 3)	69 (5, 3)			0.719

MSP = musculoskeletal pain; OR = the odds ratio; 95% CI = 95% Confidence Interval.

**Table 4:** Multivariable regression analysis of factors associated with MSP in at least one body site at any time.

Variable	*B*	*P* value	OR (95% CI)
Preclinical versus clinical			
Preclinical			
Clinical	0.278	0.395	1.32 (0.696–2.51)
History of trauma			
Yes	0.651	0.016	1.92 (0.87–4.23)
No			
History of depressive symptoms			
Yes	0.855	0.002	2.35 (1.36–4.67)
No			
History of psychosomatic symptoms			
Yes	0.99	0.004	2.69 (1.37–5.27)
No			

*B* = regression estimate; OR = the odds ratio; 95% CI = 95% Confidence Interval.
